# Autonomous Resonance‐Tuning Mechanism for Environmental Adaptive Energy Harvesting

**DOI:** 10.1002/advs.202205179

**Published:** 2022-11-28

**Authors:** Dong‐Gyu Lee, Joonchul Shin, Hyun Soo Kim, Sunghoon Hur, Shuailing Sun, Ji‐Soo Jang, Sangmi Chang, Inki Jung, Sahn Nahm, Heemin Kang, Chong‐Yun Kang, Sangtae Kim, Jeong Min Baik, Il‐Ryeol Yoo, Kyung‐Hoon Cho, Hyun‐Cheol Song

**Affiliations:** ^1^ Electronic Materials Research Center Korea Institute of Science and Technology (KIST) Seoul 02792 Republic of Korea; ^2^ Materials Science and Engineering Korea University Seoul 02841 Republic of Korea; ^3^ Department of Physics Inha University Incheon 22212 Republic of Korea; ^4^ KU‐KIST Graduate School of Converging Science and Technology Korea University Seoul 02841 Republic of Korea; ^5^ Department of Nuclear Engineering Hanyang University Seoul 04763 South Korea; ^6^ School of Advanced Materials Science and Engineering Sungkyunkwan University (SKKU) Suwon 16419 Republic of Korea; ^7^ KIST‐SKKU Carbon‐Neutral Research Center Sungkyunkwan University (SKKU) Suwon 16419 Republic of Korea; ^8^ School of Materials Science and Engineering Kumoh National Institute of Technology Gumi Gyeongbuk 39177 Republic of Korea

**Keywords:** adaptive clamps, autonomous resonance‐tuning, energy harvesting, piezoelectric, tuning beam

## Abstract

An innovative autonomous resonance‐tuning (ART) energy harvester is reported that utilizes adaptive clamping systems driven by intrinsic mechanical mechanisms without outsourcing additional energy. The adaptive clamping system modulates the natural frequency of the harvester's main beam (MB) by adjusting the clamping position of the MB. The pulling force induced by the resonance vibration of the tuning beam (TB) provides the driving force for operating the adaptive clamp. The ART mechanism is possible by matching the natural frequencies of the TB and clamped MB. Detailed evaluations are conducted on the optimization of the adaptive clamp tolerance and TB design to increase the pulling force. The energy harvester exhibits an ultrawide resonance bandwidth of over 30 Hz in the commonly accessible low vibration frequency range (<100 Hz) owing to the ART function. The practical feasibility is demonstrated by evaluating the ART performance under both frequency and acceleration‐variant conditions and powering a location tracking sensor.

## Graphical abstract

To maximize the harvesting power, the resonance frequency of the energy harvester must match that of ambient vibration. The novel autonomous resonance‐tuning (ART) energy harvester that automatically adjusts its resonance frequency in accordance with ambient vibrations under low‐frequency (<100 Hz) without any external assistance or human intervention is proposed for the first time.

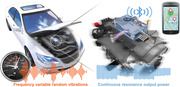

## 1 Introduction

With the advent of the era of the Fourth Industrial Revolution, the Internet of Things (IoT) combined with big data analytics will play a variety of roles in a wide range of applications, from cutting energy usage to making manufacturing safer.^[^ ^1^, ^2^, ^3^, ^4^ ^]^ The IoT is a network based on wireless sensor nodes that provide resources and information. A large number of sensors should be spread and installed over a wide range to procure sufficient data. In this situation, a critical problem arises from sensor maintenance for the viewpoint of power sources. For the long‐lasting use of IoT devices, their batteries should be replaced, or a number of power line connections with complex wiring are required. Replacing batteries or connecting long power lines for numerous sensors installed in geographically inaccessible areas is time‐consuming, dangerous, and costly. These tasks may be impossible in harsh environments, such as the outside walls of skyscrapers, deep undersea, and expansive forests.^[^ ^5^, ^6^, ^7^ ^]^ Energy harvesting technology that captures unused ambient energy and converts it into usable electrical power can provide the most feasible solution for this problem.^[^ ^8^, ^9^, ^10^ ^]^

Among ambient energies, mechanical energy is commonly available around us, particularly in industrial sites, transportation systems, and household appliances, and has a relatively higher energy density than other energy sources. Additionally, unlike solar cells, mechanical energy harvesters are not significantly affected by indoor/outdoor conditions and can operate well under harsh environments, such as dusty outdoors and rough industrial sites. There are several mechanical‐to‐electrical energy conversion scenarios, such as piezoelectric, electromagnetic, and triboelectric conversions.^[^ ^11^, ^12^, ^13^, ^14^ ^]^ Among these conversion methods, piezoelectric conversion has been the most extensively investigated for energy harvesting owing to its high energy conversion efficiency, high energy density, simple integration with vibrating platforms, and compatibility with continuous vibrations.^[^ ^15^, ^16^ ^]^

Piezoelectric energy harvesters must operate at their resonance states to maximize electrical power generation under ambient vibrations. Generally, piezoelectric transducers exhibit a narrow resonance bandwidth (less than 2 Hz). However, available ambient vibrations have a wide range of frequencies (from several Hz to hundreds of Hz), which cause frequency mismatch problems between ambient vibrations and the natural frequency of the energy harvester. Therefore, given the variable frequency conditions in real‐world implementations, it is crucial to adjust the natural frequency of the energy harvester in accordance with the surrounding vibrations for mechanical resonance. However, adjusting the natural frequency of the energy harvester by human intervention is a non‐trivial and inefficient time‐consuming task, which is one of the most critical factors preventing the commercialization of piezoelectric energy harvesters.

To address the resonance tuning problem, researchers have proposed a number of techniques, such as resonance bandwidth broadening and active resonance frequency tuning of the energy harvester.^[^ ^17^, ^18^, ^19^, ^20^, ^21^, ^22^, ^23^, ^24^, ^25^, ^26^ ^]^ The easiest way to achieve a wideband resonance frequency is by arraying energy harvesters with graded natural frequencies.^[^ ^17^, ^18^, ^19^ ^]^ However, the energy harvester array exhibits a very low power density because only one energy harvester in the array can operate in the resonance mode at a given frequency. Furthermore, a plurality of harvesters is required to create a wide resonance band; therefore, the same amount of impedance matching loads is necessary. Considering these, impedance matching with the less resistor regardless of frequency change would be a huge advantage in terms of circuitry. Another approach to obtaining the broadband resonance frequency exploits the structure's nonlinear characteristics, which exhibit wideband resonance by Duffing oscillation.^[^ ^20^ ^]^ The nonlinear system can be readily created by introducing nonlinear stiffness in the structure by applying external magnetic and mechanical forces.^[^ ^21^, ^22^, ^23^ ^]^ However, such nonlinear systems exhibit bi‐stable characteristics, such as frequency orbit jumping or hysteresis, depending on the frequency sweep direction or acceleration. Thus, this method remains challenging as a sure solution for broadening the bandwidth.^[^ ^24^ ^]^ There have also been many studies on electrical active resonance‐tuning technology, in which a sensor detects resonance and a controller adjusts the natural frequency through modulating beam stiffness as applying force by a motor.^[^ ^25^ ^]^ It is obvious that all electrical active tuning methods consume additional energy, reducing the net power output. Sometimes, the power required for frequency tuning could be greater than the harvested power.^[^ ^26^ ^]^ In other words, the electrical tuning method has a severe problem in real‐life applications owing to its low efficiency.

In this study, we present a breakthrough in piezoelectric energy harvesting by demonstrating an innovative autonomous resonance‐tuning (ART) mechanism based on an adaptive clamping system driven solely mechanically using ambient vibrations. Because only the dynamics of mechanical vibrations operate the ART system without electrical devices such as sensors, no additional energy or human intervention is required. Moreover, because a typical cantilever structure is employed, there is almost no reduction in the output power by preventing the deterioration of the quality factor. The ART energy harvester consists of a main piezoelectric energy harvester with a cantilever beam structure and several adaptive clamps that can move across the main beam harvester to adjust its clamping position. At the end of each adaptive clamp, there is a small tuning cantilever beam that provides a pulling force to move the adaptive clamp with a large centrifugal force by resonant vibration at a specific frequency; therefore, the main beam is clamped. When the vibration of a specific frequency is applied, the clamping position of the MB changes autonomously by matching the natural frequencies of the tuning beam (TB) of one of the adaptive clamps and main beam (MB). The ART energy harvester exhibits an ultrawide bandwidth of more than 30 Hz in the low‐frequency region (<70 Hz) through the self‐tuning function. Systematic design and characterization were conducted to optimize the performance of the ART energy harvester, such as maximizing the pulling force of TBs and controlling the gap size of the adaptive clamp slot for tolerance. The scientific approach for self‐tuning presented here provides a clear direction for vibration energy harvesting systems.

## 2 Results and Discussion

### 2.1 Operation Mechanism

**Figure 1:**
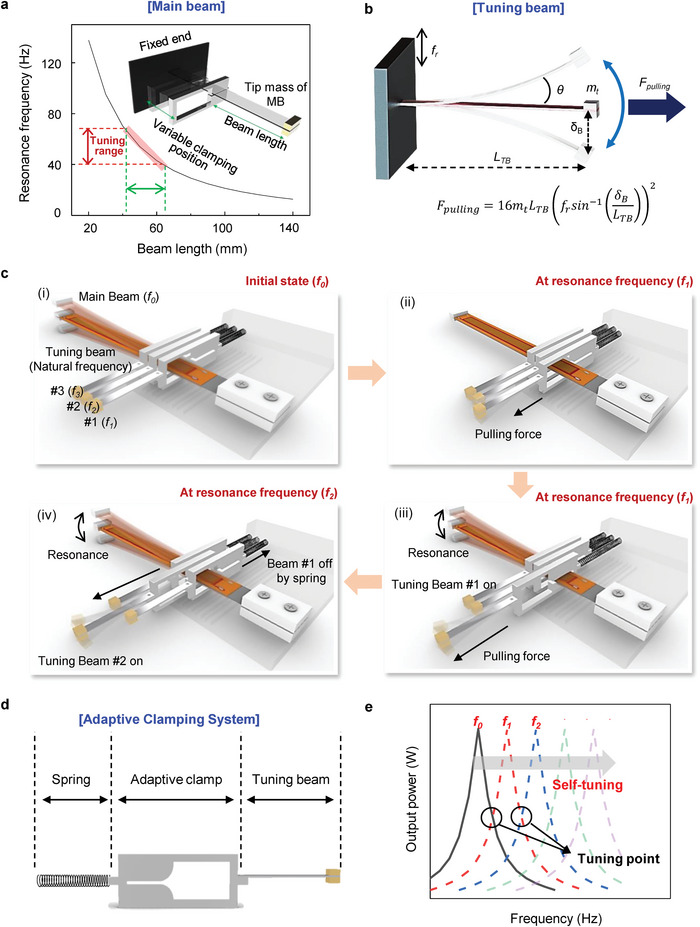
Schematic explanation of autonomous resonance‐tuning mechanism using adaptive clamping systems. a) Resonance frequency (natural frequency) of the cantilever energy harvester as a function of the beam length. b) Horizontal pulling force induced by the tuning beam vibrating at resonance state. c) Operation mechanism of autonomous resonance‐tuning function exploiting the adaptive clamping systems: (i) Initial state of harvester device in resonance state of the main beam at *f_0_*. (ii) Adaptive clamp #1 begins to move by the pulling force induced by the resonance vibration of the tuning beam #1 at *f*_1_ resonance frequency. (iii) Main beam becomes a resonance state at *f*_1_, as the adaptive clamp #1 clamps the main beam. (iv) Adaptive clamp #1 returns to its original position by a spring as it loses its pulling force with the changing vibration frequency from *f*_1_ to *f*_2_. Subsequently, adaptive clamp #2 moves and clamps the main beam at *f*_2_. d) Schematic diagram of the adaptive clamping system consisting of a spring, an adaptive clamp, and a tuning beam. e) Schematic of broadband energy harvesting via self‐tuning function of the adaptive clamping systems.

The piezoelectric energy harvester designed in this study adopted a typical cantilever structure, which is efficient and straightforward. The natural frequency of the cantilever can be derived from the Euler–Bernoulli beam equation: The first mode natural frequency (*f*_n_) of the cantilever beam can be expressed as

(1) $$f_{n}=\frac{1}{2\pi }\sqrt{\frac{3EI}{L^{3}\left(m+0.24m_{b}\right)}}$$

where *L, EI, m*, and *m*_b_ indicate the length of the cantilever beam, bending stiffness of the cantilever beam, weight of the tip mass, and mass of the cantilever beam, respectively. From Equation (1), the natural frequency of the cantilever energy harvester can be modulated by changing the weight of the tip mass, bending stiffness and length of the cantilever beam. The bending stiffness of a composite cantilever beam composed of a piezoelectric layer and a substrate layer is determined by the elastic modulus and thickness of each layer (Figure S1, Supporting Information). To tune the natural frequency, adjusting the tip mass is not an appropriate approach because it requires human intervention for the mass change.^[^ ^27^, ^28^ ^]^ In our proposed mechanism, the natural frequency of the cantilever can be readily adjusted by changing the length of the cantilever via the relocation of the clamping position, as shown in **Figure** **1**a. Meanwhile, as shown in Figure 1b, when the beam vibrates in the primary (first bending) mode, the cantilever beam generates a pulling force outward owing to the centrifugal force. The centrifugal force significantly increases at resonance because it is proportional to the displacement magnitude at the end (*δ*_B_). We designed a novel ART energy harvester based on these two basic principles: (1) the clamping position can modulate the natural frequency of the harvester main beam (MB), and (2) the tuning beam (TB) generates a large centrifugal force at resonance.

Figure 1c shows a schematic of the design of the ART energy harvester. The ART energy harvester consists of one energy‐harvesting MB and multiple adaptive clamping systems. The MB is a typical piezoelectric cantilever with a macrofiber composite piezoelectric layer for steady power generation, even if the clamping position changes. Each adaptive clamping system comprises a TB, spring, and adaptive clamp, as shown in Figure 1d. The adaptive clamp has a slot to allow the MB to be clamped. The spring and TB are connected at both ends of the adaptive clamp to provide the mechanical force to move the adaptive clamp in both directions. The TB is used to provide the pulling force to the adaptive clamp without generating electrical power. The adaptive clamping systems were placed across the MB, as illustrated in Figure 1c. The adaptive clamps can slide across the MB to modulate the clamping position of the MB.

Figure 1c,e shows the operation mechanism of the ART energy harvester by exploiting adaptive clamping systems. For instance, three adaptive clamping systems were arrayed across the MB at different positions (Figure 1c,i). The initial natural frequency of MB is *f*_0_, and the natural frequencies of TB #1, TB #2, and TB #3 are *f*_1_, *f*_2_, and *f*_3_, respectively. The modulated natural frequencies of MB sequentially increase from *f*_1_ to *f*_3_ as the operating clamping system changes from #1 to #3. This is attributed to the fact that the length of the vibrating MB decreases as the clamping position moves toward the tip mass of the MB. The natural frequencies of the TBs are designed to match the modulated natural frequencies of the MB when the adaptive clamps are installed on the MB. At *f*_0_, the MB represents its initial resonance state. If the vibration frequency changes from *f*_0_ to *f*_1_, MB and TB#1 become off‐resonance and resonance states, respectively. TB #1 vibrates significantly at *f*_1_ and drags adaptive clamp #1 by a pulling force (Figure 1c,ii). Then, adaptive clamp #1 clamps the MB, resulting in the MB being under a resonance state again at *f*_1_ (Figure 1c,iii). If the vibration frequency changes from *f*_1_ to *f*_2_, TB #1 becomes an off‐resonance state. Consequently, adaptive clamp #1 returns to its original position by a spring as the pulling force is lost. Similarly, as TB #2 becomes a resonance state at *f*_2_, adaptive clamp #2 operates and brings the MB to another resonance state at *f*_2_ (Figure 1c,iv). The tuning point (resonance frequency crossing point) (Figure 1e), which is closely related to the resonance bandwidth of the TBs, signifies the timing when the pulling force of the TB becomes greater than the sum of the frictional force by the weight of the adaptive clamp and spring force. Additionally, the bending mode of the MB was investigated by changing the clamping position of the MB and measuring the displacement distribution as shown in Figure S2 in the Supporting Information. The first bending mode was dominant in the vibrating MB. Therefore, it is possible to demonstrate the ART energy harvester without additional energy outsourcing, and the resonance bandwidth of the piezoelectric energy harvester can be significantly increased by employing multiple adaptive clamping systems.

### 2.2 Gap Size Effect in Adaptive Clamps

**Figure 2:**
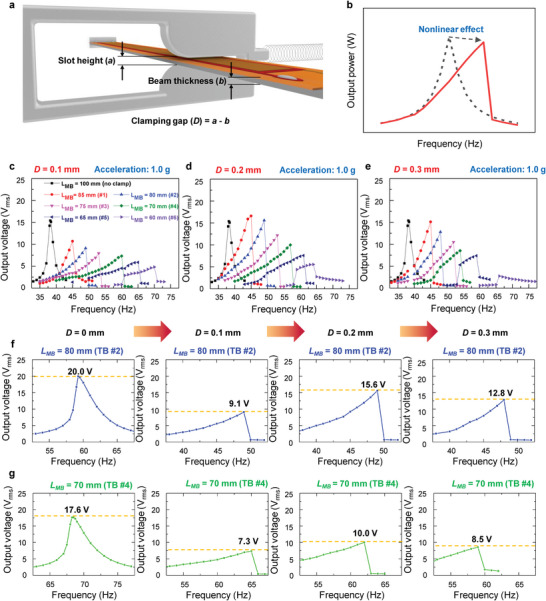
Nonlinear effect induced by slot tolerance (clamping gap) in adaptive clamps. a) Schematic illustration for the adaptive clamp slot mounted on the main beam. b) Resonance frequency broadening phenomenon by nonlinear effect observed in energy harvesters with constrained stopper. Output voltage versus vibration frequency curves of harvester main beam clamped by adaptive clamps with different clamping gaps (*D*) according to main beam length (*L*_MB_): c) *D* = 0.1 mm, d) *D* = 0.2 mm, and e) *D* = 0.3 mm. Output voltage versus vibration frequency curves of main beam for f) *L*_MB_ = 80 mm and g) *L*_MB_ = 70 mm cases with *D* = 0, 0.1, 0.2, and 0.3 mm conditions.

**Figure** **2**a shows a schematic illustrating the MB clamped by the slot of the adaptive clamp after the self‐tuning operation. For a smooth clamping process by the movement of the adaptive clamp, there must be some tolerance or gap between the slot height and thickness of the MB. The clamping gap between the MB and slot can induce a nonlinear phenomenon for the frequency response of the MB energy harvester, as shown in Figure 2b, because the clamping gap acts as a constrained stopper.^[^ ^29^, ^30^ ^]^ The nonlinear effect by the adaptive clamp gap can be described by the mathematical model employing the lumped‐parameter method as shown in Figure S3 in the Supporting Information. The MB is simplified as an equivalent mass‐spring damping model in the simulation.^[^ ^31^ ^]^ This nonlinear effect can help widen the frequency bandwidth of the ART energy harvester; thus, we evaluated the nonlinear behavior of the ART energy harvester according to the gap size in detail to determine the optimal gap size for the best operating conditions.

Figure 2c–e shows the open‐circuit output voltage of the MB energy harvester as a function of the vibration frequency with various clamping positions (i.e., various MB lengths). The position of the adaptive clamp was modulated to vary the length of the vibrating MB (*L*_MB_) from 85 to 60 mm with 5 mm intervals. The clamping gap (*D*) of the adaptive clamp was varied as *D* = 0.1, 0.2, and 0.3 mm. The acceleration of the applied vibration was 1 g ( = 9.8 m s^−2^). As shown in Figure 2c–e, resonance frequency broadening is clearly observed under all clamping gap conditions. The loose clamp provided the function of a constrained stopper, inducing a nonlinear stiffness of the MB. We found that a wider bandwidth was obtained as the *L*_MB_ decreased; however, the maximum voltage declined owing to the reduced active area by the shortened *L*_MB_. The detailed clamping gap size effect with the variation of acceleration (0.5, 1.0, and 1.5 g) in each adaptive clamping position are presented in Figure S4 in the Supporting Information. As the acceleration increased, the output voltage increased; however, the resonance frequency broadening phenomenon did not significantly depend on the change in acceleration.

Figure 2f shows the output voltage of the MB as a function of the vibration frequency for the 80 mm *L*_MB_ case with *D* = 0, 0.1, 0.2, and 0.3 mm conditions. As *D* increases, the resonance frequency decreases. This is because the larger the *D*, the looser the clamp, causing an effect as if the *L*_MB_ increased. This phenomenon was more evident for the shorter *L*_MB_ case (*L*_MB_ = 70 mm), as shown in Figure 2g. We found that an optimized gap size for the best output voltage exists in this study. As shown in Figure 2f,g, and Figure S4 (Supporting Information), the maximum output voltage of the MB was highest when *D* = 0.2 mm regardless of the acceleration magnitude and *L*_MB_. For the *D* = 0.3 mm case, the natural frequency modulations in the shortened MB were difficult, especially under the low acceleration condition (Figure S4g, Supporting Information). Therefore, the *D* = 0.2 mm condition was applied to all adaptive clamps for the best output voltage and facile natural frequency modulation.

### 2.3 Design of Tuning Beams

**Figure 3:**
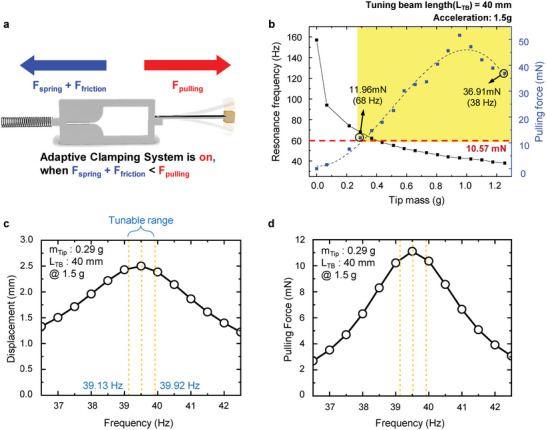
Pulling force of tuning beams. a) Schematics for three forces involved in the operation of the adaptive clamping system. The adaptive clamp system operates when the pulling force (*F*_pulling_) exceeds the sum of the spring force (*F*_spring_) and friction force (*F*_friction_). b) Pulling force and resonance frequency of tuning beam with the variation of the tip mass weight at 40‐mm‐long tuning beam length. c,d) The tuning operation range of the TB #1 was found to be between 39.13 and 39.92 Hz by calculating a displacement and pulling force at 0.29 g tip mass, 40‐mm‐long tuning beam, and 1.5 g acceleration, respectively.

In this study, the pulling force of the TB in an adaptive clamping system is a critical factor in the operation of a mechanically driven ART system. As shown in **Figure** **3**a, the spring force (*F*_spring_), friction force by the clamp weight (*F*_friction_), and pulling force by the vibration of the TB (*F*_pulling_) are involved in the operation of the adaptive clamping system. The adaptive clamp can move to modulate the natural frequency of the MB when the following relationship is satisfied

(2) $$F_{\mathrm{spring}}+F_{\mathrm{friction}}<F_{\mathrm{pulling}}$$

The spring force and friction force between the adaptive clamp and outer packaging can be expressed as

(3) $$F_{\mathrm{spring}}=k_{s}x$$

(4) $$F_{\mathrm{friction}}=\mu m_{c}g$$

where *k*_s_ *, x, µ, m*_c_, and *g* are the spring constant, extended length of spring, friction coefficient, weight of the adaptive clamp, and gravitational acceleration, respectively. The values of these forces can be easily obtained from the material constants of the components.

For the ART operation, even under low‐acceleration vibration conditions, it is necessary to maximize the *F*_pulling_ of the TB at a given frequency while minimizing *F*_spring_ and *F*_friction_. Under harmonic excitation for the base vibration at the first‐mode natural frequency of the TB, it exhibits a bending motion (Figure 3a). Assuming that the trajectory of the TB‐end is a circular arc, the centrifugal force (*F*_centrifugal_) of the TB can be expressed as

(5) $$F_{\mathrm{centrifugal}}=\frac{m_{t}v^{2}}{L_{\mathrm{TB}}}$$

where 𝑣 is the average linear speed of the TB‐end during a single period, *m*_t_ the weight of the tip mass of TB, and *L*_TB_ the length of the TB. The 𝑣 can be expressed by

(6) $$v=f_{r}d$$

where *f*_r_ is the bending‐resonance frequency (the first‐mode natural frequency) of TB and 𝑑 is the moving distance of the TB‐end (or tip mass) along the trajectory curve during a single period. The *d* can be expressed as follows

(7) $$d=4L_{\mathrm{TB}}\theta$$

Assuming that the vertical displacement (*δ*_B_ in Figure 1b) of the TB‐end is a straight line under small *θ* conditions, *θ* can be obtained as follows

(8) $$\theta =\sin^{-1}\left(\frac{\delta_{B}}{L_{\mathrm{TB}}}\right)$$

From Equations (6) to (8), 𝑣 is derived as

(9) $$v=4f_{r}L_{\mathrm{TB}}\sin^{-1}\left(\frac{\delta_{B}}{L_{\mathrm{TB}}}\right)$$

Substituting Equation (9) into Equation (5), we obtain the approximate *F*_pulling_ of the TB in the horizontal direction as follows (Figure 1b)

(10) $$F_{\mathrm{pulling}}\approx F_{\mathrm{centrifugal}}=16m_{t}L_{\mathrm{TB}}{\left(f_{r}\sin^{-1}\left(\frac{\delta_{B}\;}{L_{\mathrm{TB}}}\right)\right)}^{2}$$

In Equation (10), *F*_pulling_ is determined by *m*_t_, *L*_TB_, *f*_r_, and *δ*_B_. Comprehensive control of these parameters was conducted to design TBs with sufficient *F*_pulling_ at given frequencies. The detailed results are presented in Figure S5 in the Supporting Information. Under the condition that the *L*_TB_ was varied from 40 to 60 mm with a 5 mm interval and the width of the TB was fixed at 3.6 mm, *F*_pulling_ was calculated using Equation (10) by measuring the *δ*_B_ of the TB‐end using a laser vibrometer. For all the given *L*_TB_ cases, *f*_r_ decreased nonlinearly with increasing *m*_t_, owing to the relation *f*_n_ ∝ *m* ^0.5^ in Equation (1), whereas *F*_pulling_ exhibited a broad peak shape similar to a Gaussian function as *m*_t_ was changed. The maximum *F*_pulling_ value tended to increase as the *L*_TB_ decreased, exhibiting the largest *F*_pulling_ of ≈50 mN when *L*_TB_ = 40 mm. The required *m*_t_ to control *f*_r_ was too heavy for a shorter *L*_TB_ of less than 40 mm; therefore, we selected *L*_TB_ = 40 mm as the optimized condition for the fabrication of the ART energy harvester. Figure 3b shows the relationship between *f*_r_ and *F*_pulling_ of the experimental results according to *m*_t_ in the *L*_TB_ = 40 mm case. To evaluate the range of *f*_r_ that satisfies Equation (2), *F*_spring_ and *F*_friction_ are calculated (Figure S6, Supporting Information), and the required *F*_pulling_ to operate the adaptive clamping system was found to be at least 10.57 mN. The yellow area in Figure 3b, where *F*_pulling_ is larger than 10.57 mN, suggests that the implementation of the ART energy harvester with a broad resonance frequency range from 38 to 68 Hz is possible by employing 40‐mm‐long TBs with various tip masses (0.2 g < *m*_t_ < 1.3 g). To verify the experimental results of correlation between *f*_r_ and *F*_pulling_, we additionally calculated *δ*_B_ and *F*_pulling_ of the TB #1 as changing frequencies at 1.5 g acceleration, 40 mm *L*_TB_, and 0.29 g *m*_t_ in Figure 3c,d. An increasing *δ*_B_ of the TB #1 enabled the operation of the adaptive clamping system because the required *F*_pulling_ was higher than 10.57 mN at the tuning operation range of *f*_r_. Given these results, the natural frequency of a TB #1 can be tuned between 39.13 and 39.92 Hz.

### 2.4 Implementation of Autonomous Resonance‐Tuning Energy Harvester

**Figure 4:**
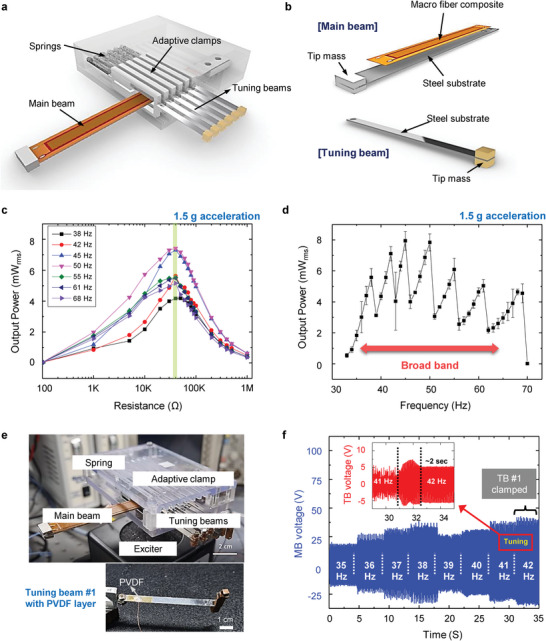
Power generating performance of the autonomous resonance‐tuning energy harvester. Schematic illustration of a) Energy harvesting device designed in this study, b) Main beam and tuning beam. c) Output power of main beam as a function of load resistance at various resonance frequencies tuned by adaptive clamping systems. The maximum power of the main beam is obtained at 40 kΩ load resistance for all resonance frequencies. d) Output power of the energy harvester at 40 kΩ load resistance measured while changing the vibration frequency. The energy harvester exhibits an ultrawide resonance bandwidth of more than 30 Hz owing to the autonomous resonance‐tuning function. e) Photographs of the fabricated energy harvester device and tuning beam #1 with a PVDF sensing layer. f) Output voltage of main beam measured while changing the vibration frequency at regular time intervals. Inset shows the voltage signal of tuning beam #1 when the main beam is clamped by adaptive clamp #1.

**Figure** **4**a shows a schematic of the designed ART energy harvester. The harvester device is composed of an MB, adaptive clamping systems, and a simple packaging case without any power‐consuming electronic circuits. Figure 4b shows the exploded‐view of the MB and TB. The MB was fabricated with a 100‐mm‐long cantilever structure composed of a piezoelectric layer (macrofibre composites, MFC) and a tip mass of 1.34 g for the initial *f*_0_ = 38 Hz. Six adaptive clamping systems were employed to broaden the operation frequency bandwidth. The natural frequencies of the six TBs were precisely adjusted by careful control of *m*_t_ to match the tuned natural frequencies of the MB. Movie S1 (Supporting Information) shows the actual operation of the ART system fabricated in this study. It was successfully demonstrated that under the condition that the vibration frequency varies from 38 to 68 Hz, the MB harvester can be continuously operated in its resonant states with a fairly large bending deformation, owing to the ART function of the adaptive clamping systems.

Impedance matching between the harvester and load resistance is essential for harvesting the maximum power from a piezoelectric energy harvester. To determine the optimum load resistance (*R*_L_) to generate the highest power under frequency‐changing conditions, we examined the output power with a variation of the connected *R*_L_ in each resonance frequency of the MB tuned by the adaptive clamps (Figure 4c). The acceleration of the vibration applied to the energy harvester was maintained at 1.5 g for all frequencies. For a vibration frequency of 38 Hz (without adaptive clamping), the output power was maximized at *R*_L_ = 40 kΩ. The *R*_L_ for the maximum power did not deviate from 40 kΩ, even when the resonance frequency was changed from 38 to 68 Hz. This is possible because only one MB is used as a harvesting component so that the inherent impedance characteristics of the harvester can be maintained even at different clamping positions. Compared to other broad‐band harvesters consisting of multiple harvesting beams with different lengths, our device has a great advantage that multiple matching resistors are not required.

Figure 4d presents the output power (at *R*_L_ = 40 kΩ) of the ART energy harvester with continuous variation of the vibration frequency under 1.5 g acceleration (*n* = 4). The seven output power peaks corresponding to without and with the adaptive clamping conditions were merged into an ultrawide resonance bandwidth of more than 30 Hz in the commonly accessible low vibration frequency range (<100 Hz), without the help of any power‐consuming frequency‐tuning device. High output power values ranging from 4.2 to 7.4 mW (power density: 3.96 µW mm^−3^)were observed at each resonance regime. More importantly, a sustainable output power of at least 2 mW can be generated at any frequency (even at tuning points) that varies in real time from 38 to 68 Hz. This phenomenon can be explained by the additional impact force exerted on the main cantilever beam (MB) by the adaptive clamp. The total output power of the energy harvester with the clamp can be higher than the harvester without the clamp because the output power generated by the impact force of the adaptive force is added to the output power generated by a resonance vibration. Additionally, when the active region becomes small enough, the output power can decrease, as shown in Figure 4d. The impact force of the clamp plays an essential role in the increased power despite the shorter active length.^[^ ^32^ ^]^

To evaluate the resonance‐frequency tuning time of the ART energy harvester, a thin polyvinylidene fluoride (PVDF) piezoelectric layer (20 µm) was attached to TB #1 (Figure 4e), and the voltage signals from the PVDF and MB were precisely monitored during the ART operation of adaptive clamp #1. As shown in Figure 4f, the TB voltage exhibited a peak when the vibration frequency reached 42 Hz and then stabilized. This peak corresponds to the largest bending deformation of TB #1 (i.e., the largest *F*_pulling_), implying the time required for the adaptive clamp #1 to start moving and clamp the MB. This tuning time was short, ≈2 s, which is consistent with the observation in Movie S1 in the Supporting Information.

Additionally, we validated the continuous power generation of the ART energy harvester under frequency variable conditions by powering 100 light‐emitting diodes (10 serial LEDs by 10 parallel LEDs) (Figure S7, Supporting Information). As shown in Movie S2 (Supporting Information), even when the vibration frequency was changed from 55 to 67 Hz in real time, the 100 LEDs were continuously and stably turned on by the ART function of TB #5 and TB #6, demonstrating the highly effective harvesting performance of the ART energy harvester over a wide frequency range. Furthermore, the piezoelectric performance of the ART energy harvester was compared to the previous studies in Table S1 in the Supporting Information.

### 2.5 Real‐World Validation of Autonomous Resonance‐Tuning Function

**Figure 5:**
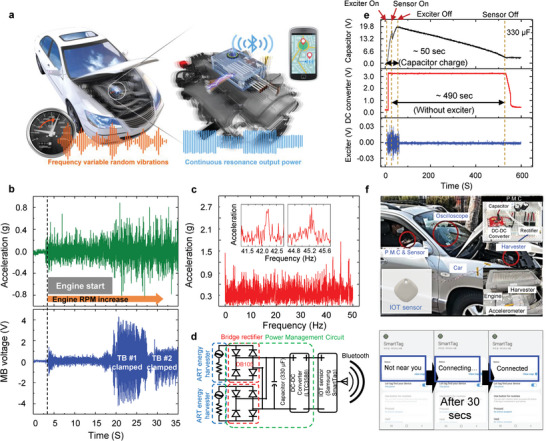
Validation of autonomous resonance‐tuning function under real‐world complex vibrations. a) Schematic image of energy harvesting and wireless sensor driving under frequency‐varying automobile engine vibration using the ART energy harvesting system. b) Real‐time variation of acceleration of engine vibration with increasing engine RPM and output voltage of main beam measured while changing the engine RPM. c) Frequency‐spectrum of acceleration amplitude for the data in (b). d) Power management circuit to provide a stable power to a wireless location tracking sensor, consisting of a bridge rectifier, a 330 µF capacitor, and a 3.3 V DC–DC converter. e) Output voltage signals from the capacitor, DC–DC converter, and vibration exciter measured to evaluate the fundamental performance of the harvesting system. The sensor can be operated for 490 s by a fully charged capacitor without a power supply. f) Experimental setup of the ART energy harvesting system mounted on a vehicle for the real‐world validation of the ART function. The wireless sensor starts working ≈30 s after the vehicle starts driving at 30 km h^−1^.

As shown in Figure 4, the ART function is stably operated over a wide range of vibration frequencies under a constant acceleration. Furthermore, we evaluated whether the ART function could be implemented under complex real‐world vibration conditions in which both acceleration and frequency were changed. An automobile engine was selected as the frequency variable random vibration source as described in **Figure** **5**a, and its vibration characteristics were examined using an accelerometer while changing the engine RPM. As shown in Figure 5b, the acceleration of the engine vibration changed randomly and complexly with increasing engine RPM. From the fast Fourier transform analysis of the result in Figure 5b, it was found that a number of vibration frequencies with various acceleration amplitudes existed in the range of 0–50 Hz (Figure 5c). Because this frequency range contained the *f_r_* of TB #1 (≈42 Hz) and TB #2 (≈45 Hz), the ART function was expected to be operated in our harvester device. Remarkably, even at accelerations much smaller than 1 g, adaptive clamps #1 and #2 successfully moved and clamped the MB sequentially (Movie S3, Supporting Information), resulting in high output voltages at the two resonance states of MB (Figure 5b).

Finally, we demonstrated the operation of a wireless location tracking sensor (Global Positioning System, Samsung SmartTag) solely powered by the ART energy harvester under the complex engine vibration. The sensor requires 3 V and 20 mW to start operating. For a stable supply of electrical power to the sensor, a power management circuit (Figure 5d) composed of a bridge rectifier (DB105), a 330 µF capacitor, and a 3.3 V DC‐DC converter (LTC3588, SparkFun) was designed. First, the fundamental performance of the power management circuit on the harvesting system for operating the sensor was evaluated under a constant vibration condition (Figure 5e; Figure S8, Supporting Information). Under the exciter vibration of 1.5 g‐acceleration at 38 Hz, the ART energy harvester fully charged the capacitor within 50 s and the sensor started working (“Sensor On” in Figure 5e) before the capacitor was fully charged. After that, the sensor stably operated for 490 s by the charged capacitor even without a vibration source (“Exciter Off” in Figure 5e). Next, the practical performance of the ART energy harvesting system for operating the sensor was verified by applying it to an automobile engine (Figure 5f). While driving a vehicle at a speed of 30 km h^−1^, the ART harvester continuously charged the capacitor and successfully operated the wireless sensor (see Movie S4 in the Supporting Information for the operation of the wireless sensor connected to a smartphone (Samsung Galaxy S21) via Bluetooth while driving a vehicle). To the best of our knowledge, this is the first time that the ART function has been implemented under real‐world vibrations in which acceleration and frequency change simultaneously. This result demonstrates the practical potential of our ART energy harvester for harvesting broadband low‐frequency (<100 Hz) vibrational energy commonly found in our surroundings.

## 3 Conclusion

In summary, we developed an innovative autonomous resonance‐tuning (ART) vibration energy harvester utilizing adaptive clamping systems driven purely by a mechanical mechanism without additional power‐consuming devices. In the proposed ART system, the resonance frequency of the harvester main beam (MB) was autonomously tuned as the vibration frequency changed, owing to the reversible adaptive clamping function operated by the pulling force of the tuning beams (TB). The clamping slot of the adaptive clamp was designed to be larger than the thickness of the MB, which induced a nonlinear stiffness of the MB, resulting in the resonance bandwidth broadening effect. The optimized gap size between the slot and MB for the best output voltage existed. Comprehensive control of the design parameters of the TB was conducted to determine the sufficient pulling force of the TB for ART operation at various frequencies, and the milestone for the TB design satisfying the required operation frequency window was presented. The energy harvester, composed of six adaptive clamping systems, successfully demonstrated the ART harvesting function in a wide vibration frequency range from 38 to 68 Hz. Only one load resistor was sufficient for impedance matching to obtain the maximum output power from the MB harvester at all resonance frequencies. The resonance‐frequency tuning time was as short as 2 s. Encouragingly, the ART function was implemented even under complex automobile engine vibrations, where both the acceleration and frequency were changed. The ART energy harvester presented in this study is expected to open a new horizon for vibration energy harvesting technology owing to its novel self‐tuning ability in frequency‐variant environments.

## 4 Experimental Section

### Fabrication of Autonomous Resonance‐Tuning (ART) Energy Harvester

To fabricate the ART energy harvester, as shown in Figure 4, a 31 mode macrofiber composite (MFC, M8507‐P2, Smart Material GmbH, Germany), stainless steel substrates (SUS304, Jaeyun‐Jungmil, Korea), adaptive clamp body (Aluminum, Jaeyun‐Jungmil, Korea), tip masses (Cu and Pb, Jaeyun‐Jungmil, Korea), and springs (Woosung Spring, Korea) were prepared and customized according to the designed dimensions. The detailed parameters of the MFC can be described by 63 nF of capacitance, −605 ppm of free strain, −60 to 360 V of operating voltage range, −210 pC N^−1^ of piezoelectric constant (*d*_31_), and −38 N of blocking force, respectively. The harvester packaging case was fabricated using acrylic material. The stainless steel substrates and acrylic harvester case were cleaned with DI water for 10 min by sonication and dried at 37 °C for 2 h. The 1‐mm‐thick harvester main beam (MB) was prepared by attaching the MFC and tip masses (Cu) on the stainless steel substrate using epoxy adhesive (DP460, 3 M, USA). The 0.3‐mm‐thick tuning beams (TB) were prepared by attaching tip masses (Cu) to the stainless steel substrate. A 20‐µm‐thick polyvinylidene fluoride (PVDF) film with a Cr/Au electrode (FC20, Piezotech, France) was purchased and attached to TB #1 for voltage signal sensing. Before assembling the harvester, electrical leads were connected to the MFC and PVDF films.

### Characterization and Measurements

To verify the voltage and power output characteristics in Figures 2 and 4, and Figure S5 (Supporting Information), a vibration was applied to the harvester device using a vibration exciter (K2007E01, The Modal Shop, USA), which was operated by a function generator (WF 1943A 1CH, NF Corporation, Japan). The open‐circuit voltage from the MB and TB #1 was monitored using an oscilloscope (DSOX1204G, Keysight, USA). The MB movement was measured by the Laser Doppler Vibrometer (Polytec, PSV‐400, Germany). The output power (*P*_out_) of the harvester was obtained by measuring the closed‐circuit voltage (*V*_cc_) from the MB using an oscilloscope after connecting a load resistor (*R*_L_) ($P_{\mathrm{out}}=V_{\mathrm{cc}}^{2}/R_{L}$). To obtain the pulling force values of TB in Figure 3b and Figure S6 (Supporting Information), the vertical displacement of the TB‐end (*δ*_B_ in Figure 1b) was measured using a scanning laser vibrometer (PSV‐400, Polytec Inc., USA). The acceleration of the automobile engine (Figure 5b) was recorded using an accelerometer (482A21, PCB Piezotronics, USA) mounted on the surface of the engine cover. For the data in Figure 5b, the ART energy harvester was mounted on the surface of the engine cover, and the output voltage from the MB was recorded using an oscilloscope.

## Conflict of Interest

The authors declare no conflict of interest.

## Supporting information

Supporting Information

Supplemental Movie 1

Supplemental Movie 2

Supplemental Movie 3

Supplemental Movie 4

## Data Availability

The data that support the findings of this study are available from the corresponding author upon reasonable request.

## References

[1] M. Shirvanimoghaddam , K. Shirvanimoghaddam , M. M. Abolhasani , M. Farhangi , V. Z. Barsari , H. Liu , M. Dohler , M. Naebe , IEEE Access 2019, 7, 94533.

[2] F. Narita , M. Fox , Adv. Eng. Mater. 2018, 20, 1700743.

[3] P. Kamalinejad , C. Mahapatra , Z. Sheng , S. Mirabbasi , V. C. M. Leung , Y. L. Guan , IEEE Commun. Mag. 2015, 53, 102.

[4] X. Zhao , H. Askari , J. Chen , Joule 2021, 5, 1391.

[5] Z. Chu , Z. Sun , B. Wang , K. Song , J. Wang , J. Gao , S. Dong , Adv. Energy Mater. 2022, 12, 2103345.

[6] Z. Yu , H. Qiu , Z. Chu , Z. Sun , M. Asl , F. Li , S. Dong , Adv. Energy Mater. 2022, 10.1002/aenm.202202306

[7] Z. Yu , J. Yang , J. Cao , L. Bian , Z. Li , X. Yuan , Z. Wang , Q. Li , S. Dong , Adv. Funct. Mater. 2022, 32, 2111140.

[8] H. Song , P. Kumar , D. Maurya , M. Kang , W. T. Reynolds , D. Jeong , C. Kang , S. Priya , J. Microelectromech. Syst. 2017, 26, 1226.

[9] S. Priya , D. J. Inman , Energy Harvesting Technologies, Springer US, Boston, MA 2009.

[10] H. Fu , X. Mei , D. Yurchenko , S. Zhou , S. Theodossiades , K. Nakano , E. M. Yeatman , Joule 2021, 5, 1074.

[11] A. Erturk , J. Hoffmann , D. J. Inman , Appl. Phys. Lett. 2009, 94, 254102.

[12] S. P. Beeby , M. J. Tudor , N. M. White , Meas. Sci. Technol. 2006, 17, R175.

[13] J. Chen , Z. L. Wang , Joule 2017, 1, 480.

[14] Z. Yang , S. Zhou , J. Zu , D. Inman , Joule 2018, 2, 642.

[15] H.‐C. Song , S.‐W. Kim , H. S. Kim , D.‐G. Lee , C.‐Y. Kang , S. Nahm , Adv. Mater. 2020, 32, 2002208.

[16] A. Toprak , O. Tigli , Appl. Phys. Rev. 2014, 1, 031104.

[17] Z. Xiao , Appl. Phys. Lett. 2014, 104, 223904.

[18] I. C. Lien , Y. C. Shu , Smart Mater. Struct. 2012, 21, 082001.

[19] K. Mikoshiba , J. M. Manimala , C. Sun , J. Intell. Mater. Syst. Struct. 2013, 24, 168.

[20] M. F. Daqaq , R. Masana , A. Erturk , D. D. Quinn , Appl. Mech. Rev. 2014, 66, 040801.

[21] H.‐C. Song , P. Kumar , R. Sriramdas , H. Lee , N. Sharpes , M.‐G. Kang , D. Maurya , M. Sanghadasa , H.‐W. Kang , J. Ryu , W. T. Reynolds , S. Priya , Appl. Energy 2018, 225, 1132.

[22] A. F. Arrieta , P. Hagedorn , A. Erturk , D. J. Inman , Appl. Phys. Lett. 2010, 97, 104102.

[23] E. Dechant , F. Fedulov , D. V. Chashin , L. Y. Fetisov , Y. K. Fetisov , M. Shamonin , Smart Mater. Struct. 2017, 26, 065021.

[24] J.‐W. Ko , Y.‐B. Bin , W.‐J. Eun , S.‐J. Shin , J. Comput. Struct. Eng. Inst. Korea 2015, 28, 467.

[25] M. Mösch , G. Fischerauer , D. Hoffmann , Sensors 2020, 20, 2519. 32365593 10.3390/s20092519 PMC7248692

[26] J. Esch , D. Hoffmann , D. Stojakov , Y. Manoli , J. Phys. Conf. Ser. 2019, 1407, 012012.

[27] D. Zhu , S. Roberts , M. J. Tudor , S. P. Beeby , Sens. Actuators, A 2010, 158, 284.

[28] E. S. Leland , P. K. Wright , Smart Mater. Struct. 2006, 15, 1413.

[29] M. S. M. Soliman , E. M. Abdel‐Rahman , E. F. El‐Saadany , R. R. Mansour , J. Micromech. Microeng. 2008, 18, 115021.

[30] H. Farokhi , M. H. Ghayesh , Energy Convers. Manage. 2019, 197, 111828.

[31] A. Erturk , D. J. Inman , J. Sound Vib. 2011, 330, 2339.

[32] K. Zhou , H. L. Dai , A. Abdelkefi , H. Y. Zhou , Q. Ni , AIP Adv. 2019, 9, 035228.

