# Supplementary material for: Autonomous Resonance‐Tuning Mechanism for Environmental Adaptive Energy Harvesting
Source: Adv Sci (Weinh). 2022 Nov 28;10(3):2205179. doi: 10.1002/advs.202205179 (PMC9875603; doi:10.1002/advs.202205179)
Supplement: Supplementary file 1 — Supporting Information [file ADVS-10-2205179-s004.pdf]

Supporting Information

Autonomous Resonance-tuning Mechanism for Environmental Adaptive Energy Harvesting

Dong-Gyu Lee[¶], *Joonchul Shin*[¶], *Hyun Soo Kim, Sunghoon Hur, Shuailing Sun, Ji-Soo Jang, Sangmi Chang, Inki Jung, Sahn Nahm, Heemin Kang, Chong-Yun Kang, Sangtae Kim, Jeong Min Baik, Il-Ryeol Yoo, Kyung-Hoon Cho*[*], *and Hyun-Cheol Song*[*]

D. -G. Lee, Dr. J. Shin, H. S. Kim, Dr. S. Hur, S. Sun, Dr. J. -S. Jang, S. Chang, Dr. I. Jung, Dr. C. -Y. Kang, H. -C. Song

Electronic Materials Research Center, Korea Institute of Science and Technology (KIST), Seoul 02792, Republic of Korea
*E-mail: hcsong@kist.re.kr

D. -G. Lee, Prof. S. Nahm, Prof. H. Kang

Materials Science and Engineering, Korea University, Seoul 02841, Republic of Korea

H. S. Kim

Department of Physics, Inha University, Incheon 22212, Republic of Korea

S. Chang, Prof. S. Nahm, Dr. C. -Y. Kang

KU-KIST Graduate School of Converging Science and Technology, Korea University, Seoul 02841, Republic of Korea

Prof. S. Kim

Department of Nuclear Engineering, Hanyang University, Seoul, South Korea

Prof. J. M. Baik, Dr. H. -C. Song

KIST-SKKU Carbon-Neutral Research Center, Sungkyunkwan University (SKKU), Suwon 16419, Republic of Korea

I. -R. Yoo, Prof. K. -H. Cho

School of Materials Science and Engineering, Kumoh National Institute of Technology, Gumi, Gyeongbuk, 39177, Republic of Korea

*E-mail: khcho@kumoh.ac.kr

[¶]These authors contributed equally.

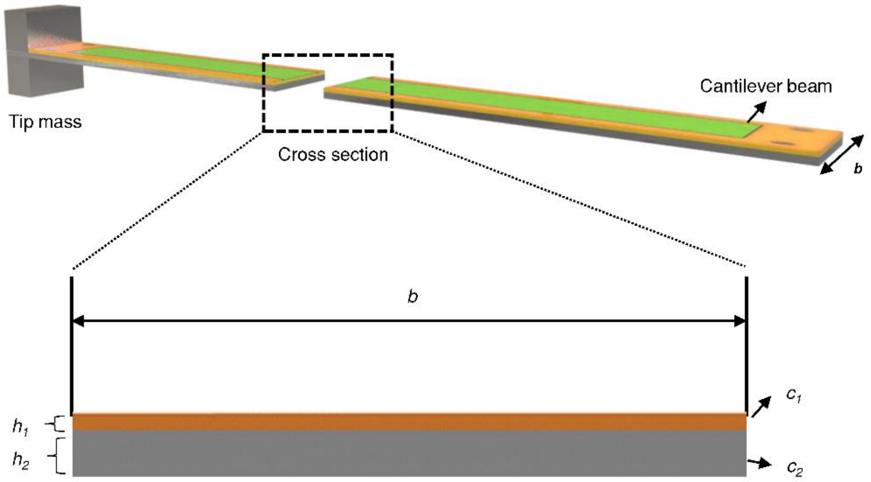

Bending stiffness[1, 2] of composite beam is defined as

$$EI = \frac{b\cdot c_1}{3}\left\{\left[\frac{c_2(h_1h_2+h_2^2)}{c_2h_2+c_1h_1}\right]^3 - \left[\frac{c_2h_2^2-c_1h_1^2}{2(c_2h_2+c_1h_1)}\right]^3\right\} +$$

$$\frac{b\cdot c_2}{3}\left\{\left[\frac{c_2h_2^2-c_1h_1^2}{2(c_2h_2+c_1h_1)}\right]^3 + \left[\frac{2c_1h_1h_2+c_1h_1^2+c_2h_2^2}{2(c_2h_2+c_1h_1)}\right]^3\right\}$$

c_1 : *Elastic modulus of piezoelectric layer*

c_2 : *Elastic modulus of substrate layer*

h_1 : *Thickness of piezoelectric layer*

h_2 : *Thickness of substrate layer*

Fig. S1. The bending stiffness (*EI*) of a composite cantilever beam composed of a piezoelectric layer and a substrate layer.

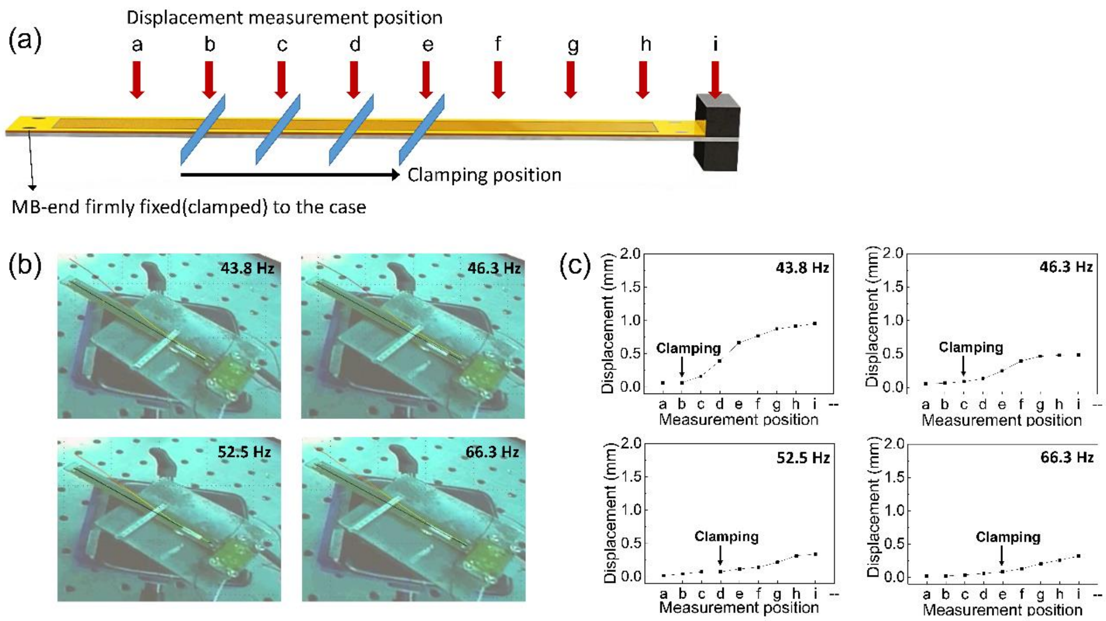

Fig. S2. Displacement distribution by shifting a clamp position on the main beam. (a) Schematic diagram of the main beam for the displacement measurement. (b) Movement of the main beam according to the change of the clamping position. (c) Displacement distribution of the main beam.

Mathematical model for nonlinear effect in adaptive clamping system

Regardless of the gravity of the main beam (MB), the governing equations of the model are derived from Newton's second law and Kirchhoff's first law.[3]

$$M_{eq}\ddot{x} + \eta_{eq}(\dot{x} - \dot{x_0}) + K_{eq}(x - x_0) + \theta U = 0 \quad (1)$$

$$\theta(\dot{x} - \dot{x_0}) = C_P\dot{U} + \frac{U}{R_L} \quad (2)$$

$$K_{eq} = \begin{cases} K_0 \left(-\frac{D}{2} < x < \frac{D}{2}\right) \\ K_1 \ (x \leq -\frac{D}{2} \ or \ x \geq \frac{D}{2}) \end{cases} \quad (3)$$

where M_{eq}, K_{eq} and η_{eq} represent the equivalent mass, equivalent stiffness and equivalent damping of the MB, respectively; C_P, θ, and R_L represent the equivalent capacitance, electromechanical coupling coefficient of the piezoelectric layer and external resistance, respectively; x and x_0 are the absolute displacement of the MB and base, respectively. The equivalent stiffness of the MB increases when it comes in contact with the clamp, which causes the nonlinearity of the structure. Fig. S2 below represents the simulated output voltage when the MB is in linear and nonlinear states, respectively. The natural frequency in the nonlinear state increases compared with that in the linear state. Additionally, the output voltage in the nonlinear state has a sharp decrease while that in the linear state decreases gradually.

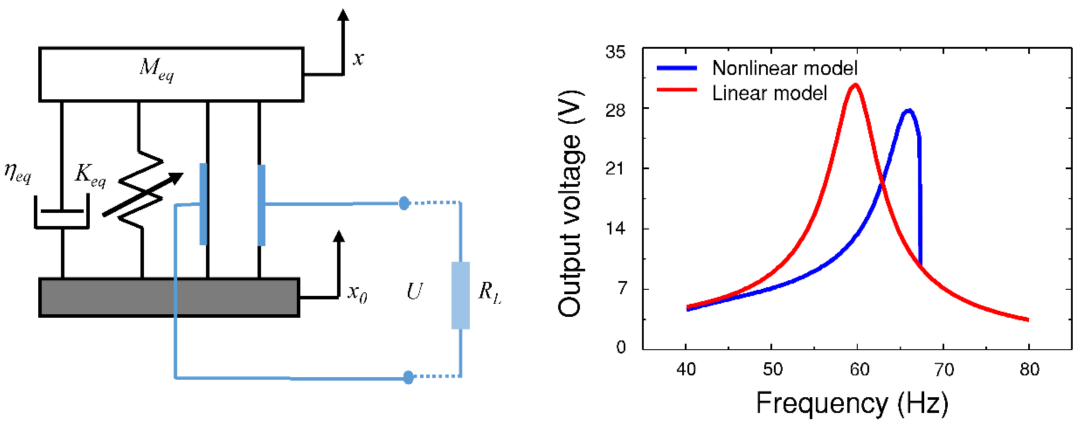

M_{eq}: Equivalent mass of the main beam (MB)
K_{eq} : Equivalent stiffness of the MB
η_{eq} : Equivalent damping of the MB
C_P : Equivalent capacitance

θ : Electromechanical coupling coefficient of the piezoelectric layer
R_L: Electromechanical coupling coefficient of the external resistance
x : Displacement of the MB
x_0 : Displacement of the base

Fig. S3. The mathematical model for nonlinear effect of the adaptive clamping system. The simulated output voltages of the MB are presented in linear and nonlinear states, respectively.

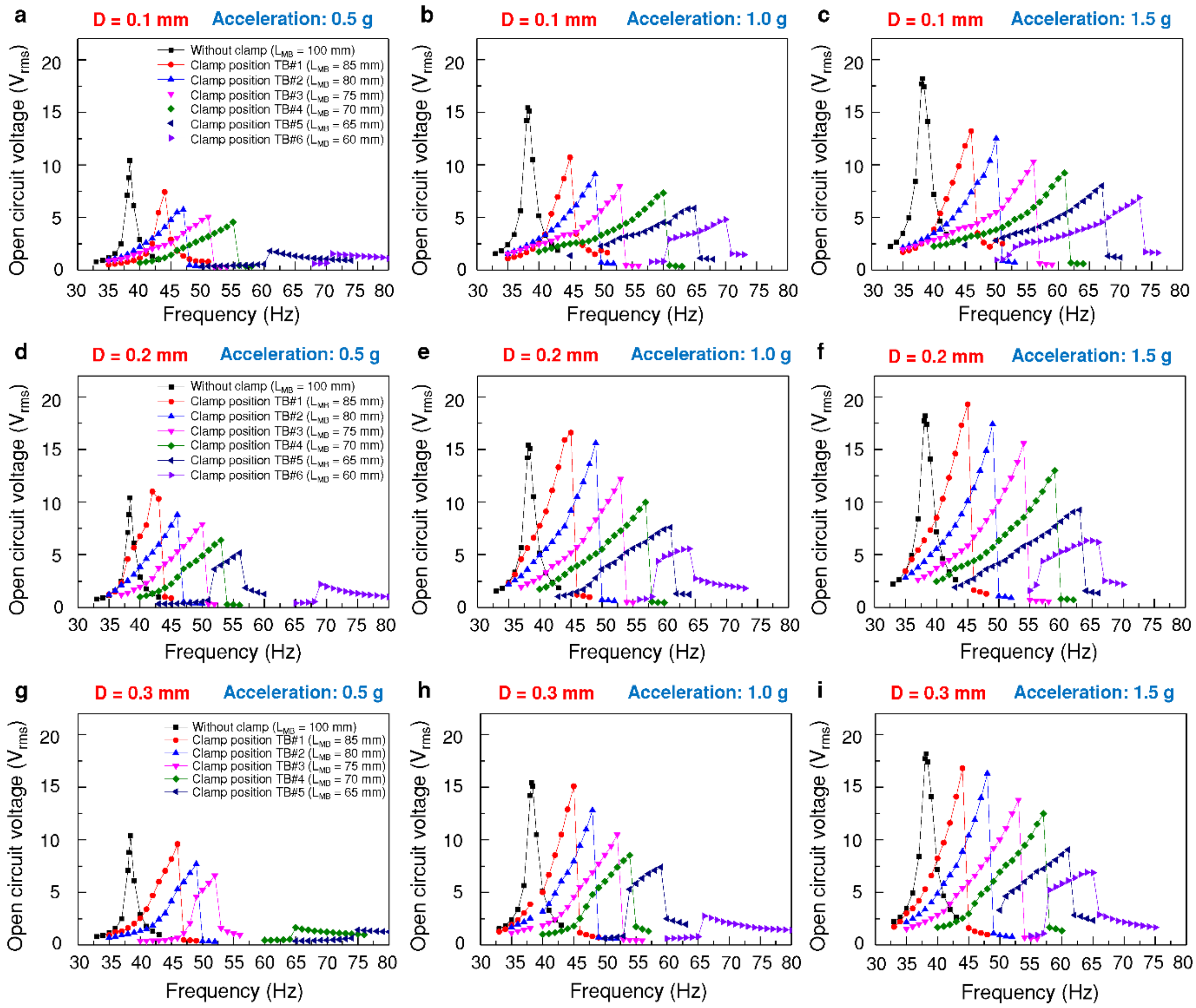

Fig. S4. Output voltage vs. vibration frequency curves of harvester main beam clamped by adaptive clamps with various clamping gap (*D*) and main beam length (L_{MB}) conditions measured at different accelerations of applied vibration.

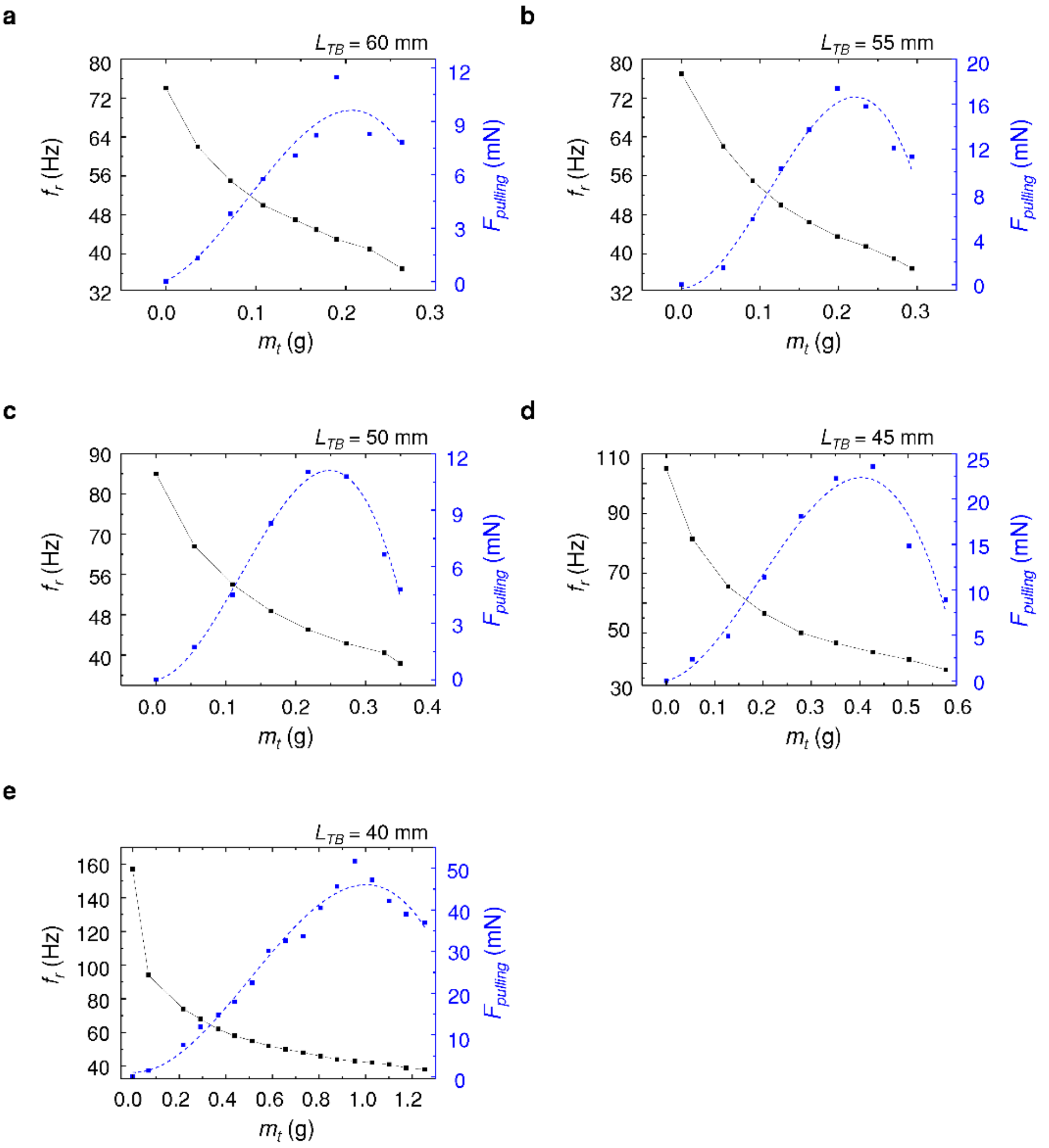

Fig. S5. Pulling force ($F_{pulling}$) and resonance frequency of tuning beam (f_r) with the variation of the tip mass weight (m_t) at various tuning beam lengths (L_{TB}) measured at 1.5 g acceleration: (a) L_{TB} = 60 mm, (b) L_{TB} = 55 mm, (c) L_{TB} = 50 mm, (d) L_{TB} = 45 mm, and (e) L_{TB} = 40 mm.

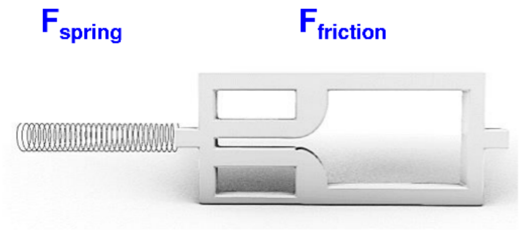

Adaptive Clamping System is on, when
$F_{spring} + F_{friction} < F_{pulling}$

1. Spring force (3.71 mN)

$$F_1 = kx$$
$$F_1 = \frac{G \cdot d^4}{8 \cdot N \cdot D^3} \cdot x$$

k : spring constant
x : displacement (1.5×10^{-2} m)
G : shear modulus of elasticity (77.2×10^{9} N/m^2)
N : number of active coils turns (266.667)
D : center diameter of coil (4.2×10^{-3} m)
d : wire diameter (1.5×10^{-4} m)

2. Friction force (6.86 mN)

$$F_2 = \mu \cdot N$$
$$F_2 = \mu \cdot m \cdot g$$

N : normal force
m : mass (3.50 g)
g : gravitational acceleration (9.8 m/s^2)
μ : coefficient of friction (0.2)

Fig. S6. Spring force and friction force of adaptive clamping system. The sum of the spring force and friction force was calculated to be 10.57 mN.

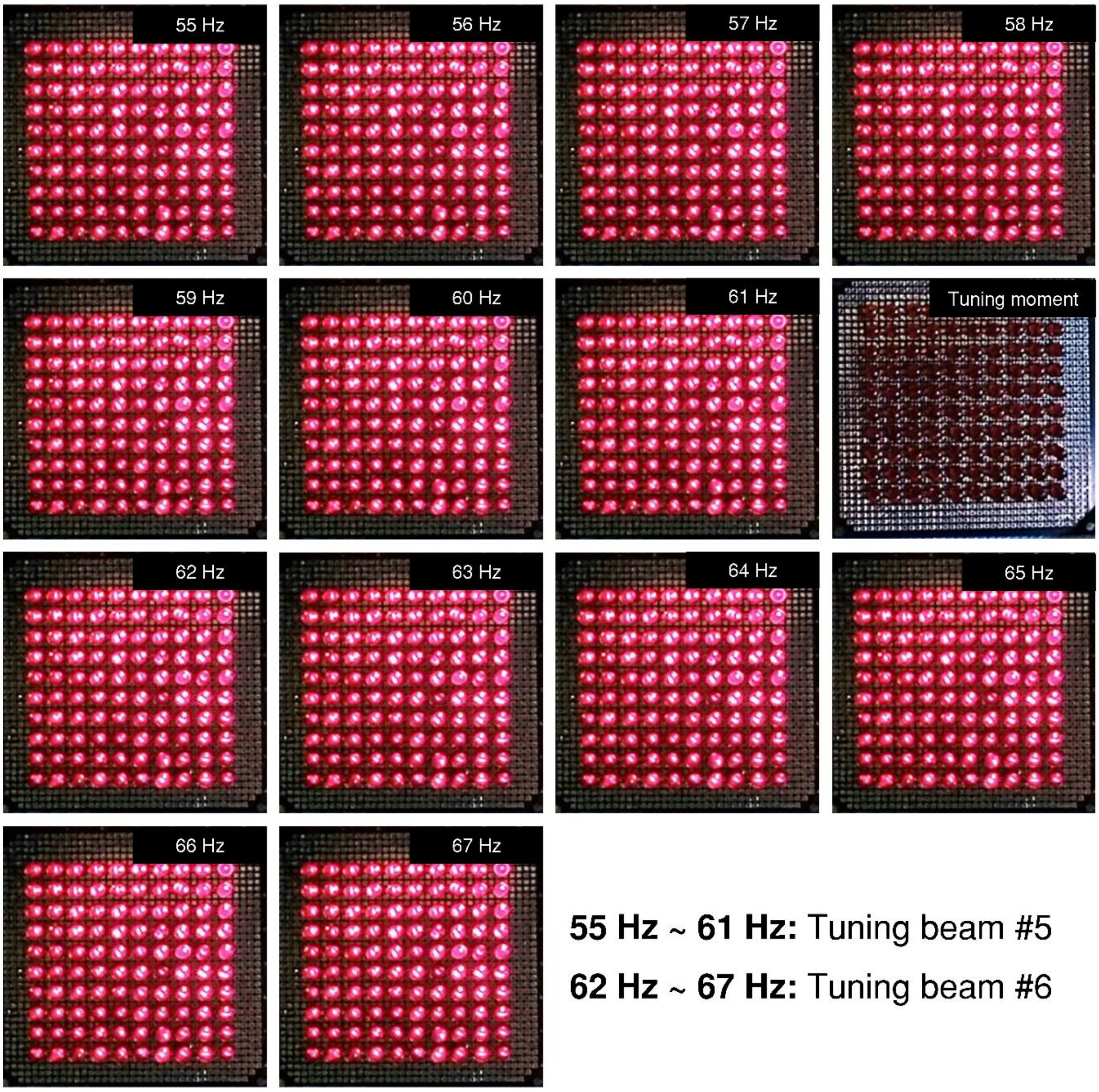

Fig. S7. 100 LEDs powered by the ART energy harvester under real-time changing vibration frequency from 55 Hz to 67 Hz. The actual operation and experimental setup can be seen in Movie S2.

Control method	Tuning range (Hz)	Untuned Natural frequency (Hz)	Tunability (%)	RMS power (μW)	Power density (μW/mm^3)	Acceleration (g)	Reference
Manual	25.5-62	33.5	109	2.78	6.95×10^{-4}	0.3	4
Manual	51-87	61	59.2	-	-	-	5
Manual	160-190	190	15.8	650	8.45	1	6
Manual	35-41	35	31	10	-	0.015	7
Manual	6-61.8	29.91	186.6	-	-	-	8
Manual	265-320	280	19.64	50	8.77	1	9
Microcontroller	91.5-94.5	92.5	3.2	3600	-	0.1	10
Microcontroller	42-52	44	30	1400	10.72	-	11
Microcontroller	106-118	112	10.7	0.45	1.44×10^{-3}	0.5	12
Microcontroller	24-45	27.5	25.5	93	1.48×10^{-5}	0.041	13
Microcontroller	49-54	53	9.4	5310	-	1	14
Autonomous	24-32	24	33.3	0.1	-	-	15
Autonomous	22-35	22	59.1	-	-	0.5	16
Autonomous	5.75-9.05	7.42	44.5	1800	3.33	0.96	17
Autonomous	0-91	45.5	100	90	2.59×10^{-2}	0.5	18
Autonomous	6.2-16.2	13.2	75.8	0.83	4.61×10^{-3}	-	19
Autonomous	38-68	38	78.95	7400	3.96	1.5	**This work**

Table S1. Properties comparison of resonance-tunable piezoelectric energy harvester

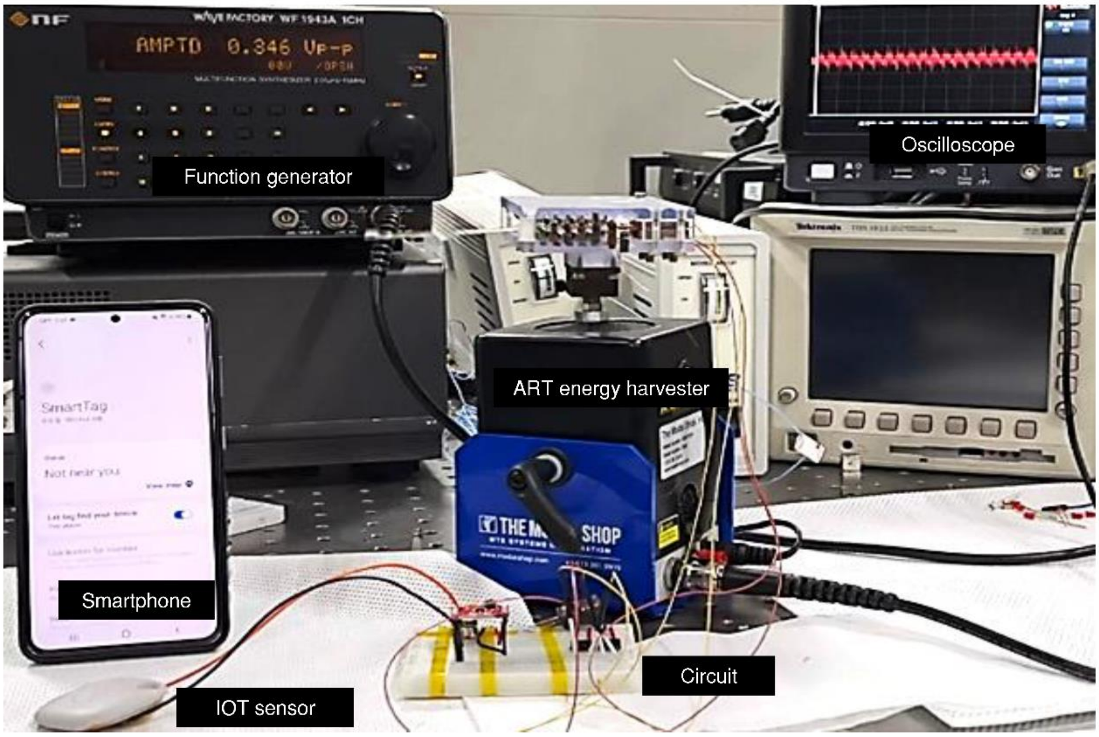

Fig. S8. Operation of the Bluetooth wireless location tracking sensor by the ART energy harvester with the power management circuit consisting of a bridge rectifier, a 330 μF capacitor, and a 3.3 V DC-DC converter.

References

1. K.-H. Cho, H.-Y. Park, J. S. Heo, S. Priya, *J. Appl. Phys.* **2014**, *115*, 204108.
2. M. Brissaud, S. Ledren, P. Gonnard, *J. Micromechanics Microengineering* **2003**, *13*, 832.
3. S. Sun, Y. Leng, X. Su, Y. Zhang, X. Chen, J. Xu, *Energy Convers. Manag.* **2021**, *239*, 114246.
4. H. Xia, R. Chen, L. Ren, *Sensor Actuators Phys.* **2017**, *257*, 73.
5. L. Dong, M. G. Prasad, F. T. Fisher, *Smart Mater. Struct.* **2016**, *25*, 065019.
6. E. S. Leland, P. K. Wright, *Smart Mater. Struct.* **2006**, *15*, 1413.
7. J. Schaufuss, D. Scheibner, J. Mehner, *Sens. Actuators Phys.* **2011**, *171*, 352.
8. E. Dehghan Niri, S. Salamone, *Smart Mater. Struct.* **2012**, *21*, 125025.
9. M. Wischke, M. Masur, F. Goldschmidtboeing, P. Woias, presented at 2010 IEEE 23rd Int. Conf. on Micro Electro Mechanical Systems, Wanchai, Hong Kong, China, January **2010**.
10. W.-J. Wu, Y.-Y. Chen, B.-S. Lee, J.-J. He, Y.-T. Peng, *Smart Structures and Materials 2006: Damping and Isolation* **2006**, p. 61690A.
11. C. Peters, D. Maurath, W. Schock, F. Mezger, Y. Manoli, *J. Micromech. Microeng.* **2009**, *19*, 094004.
12. M. Lallart, S. R. Anton, D. J. Inman, *J. Intell. Mater. Syst. Struct.* **2010**, *21*, 897.
13. A. Brenes, A. Morel, D. Gibus, C.-S. Yoo, P. Gasnier, E. Lefeuvre, A. Badel, *Sens. Actuators Phys.* **2020**, *302*, 111759.
14. C. V. Karadag, N. Topaloglu, *J. Vib. Acoust.* **2017**, *139*, 011013.
15. S.-E. Jo, M.-S. Kim, Y.-J. Kim, *Smart Mater. Struct.* **2012**, *21*, 015007.
16. R. Somkuwar, J. Chandwani, R. Deshmukh, *Microsyst. Technol.* **2018**, *24*, 3033.
17. H. Kim, L. Zuo, W. che Tai, in *Activeand Passive Smart Structures and Integrated Systems XII* (Ed: A. Erturk), SPIE, Denver, CO **2018**, p. 29.
18. F. Qian, S. Zhou, L. Zuo, *Commun. Nonlinear Sci. Numer. Simul.* **2020**, *80*, 104984.
19. L. Gu, C. Livermore, *Appl. Phys. Lett.* **2010**, *97*, 081904.
